# Fabry Disease and Inflammation: Potential Role of p65 iso5, an Isoform of the NF-κB Complex

**DOI:** 10.3390/cells14030230

**Published:** 2025-02-06

**Authors:** Giuseppa Biddeci, Gaetano Spinelli, Paolo Colomba, Giovanni Duro, Monia Anania, Daniele Francofonte, Francesco Di Blasi

**Affiliations:** Institute for Biomedical Research and Innovation, National Research Council of Italy (IRIB-CNR), Via Ugo La Malfa 153, 90146 Palermo, Italy; giuseppa.biddeci@irib.cnr.it (G.B.); gaetano.spinelli@irib.cnr.it (G.S.); paolo.colomba@irib.cnr.it (P.C.); giovanni.duro@irib.cnr.it (G.D.); monia.anania@irib.cnr.it (M.A.); daniele.francofonte@irib.cnr.it (D.F.)

**Keywords:** Fabry disease, inflammation, NF-κB, p65, p65 iso5

## Abstract

Fabry disease (FD) is an X-linked lysosomal storage disease, caused by mutations in the *GLA* gene on the X chromosome, resulting in a deficiency of the lysosomal enzyme α-GAL. This leads to the progressive accumulation of Gb3 in cells, causing multi-systemic effects. FD has been classified as a subgroup of autoinflammatory diseases. NF-κB is a family of ubiquitous and inducible transcription factors that play critical roles in inflammation, in which the p65/p50 heterodimer is the most abundant. The glucocorticoid receptor (GR) represents the physiological antagonists in the inflammation process. A novel spliced variant of p65, named p65 iso5, which can bind the dexamethasone, enhancing GR activity, has been found. This study investigates the potential role of p65 iso5 in the inflammation of subjects with FD. We evaluated in peripheral blood mononuclear cells (PBMCs), from over 100 FD patients, the p65 iso5 mRNA level, and the protein expression. The results showed significantly lower p65 iso5 mRNA and protein expression levels compared to controls. These findings, along with the ability of p65 iso5 to bind dexamethasone and the regulation of the glucocorticoid response in the opposite way of p65, strongly suggest the involvement of p65 iso5 in the inflammatory response in FD.

## 1. Introduction

**Figure 1:**
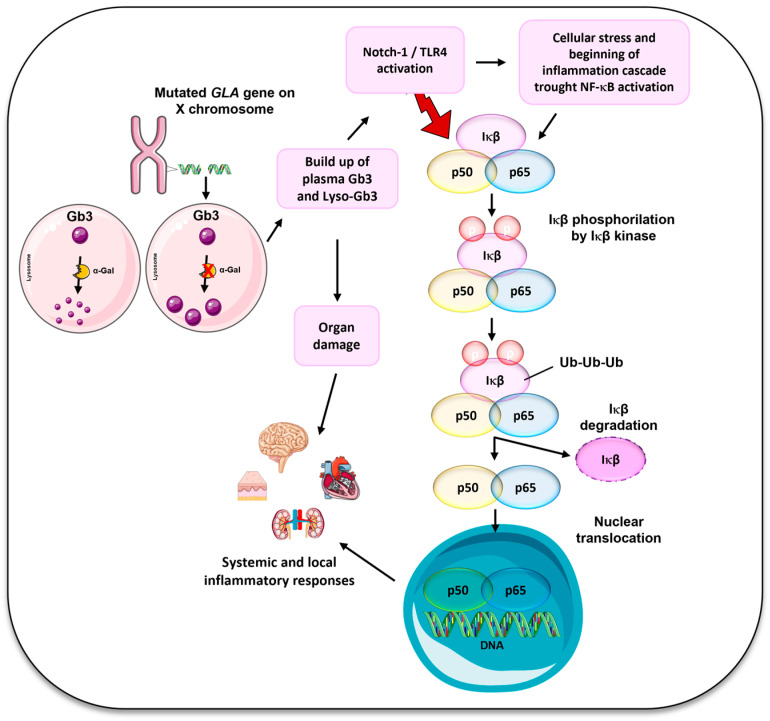
Molecular mechanisms in inflammation related Fabry disease. Mutations in the *GLA* gene on the X chromosome lead to deficient alpha-galactosidase A (α-Gal) enzyme activity. This results in the accumulation of globotriaosylceramide (Gb3) and its deacylated form Lyso-Gb3 within lysosomes. The Notch-1/TLR4 activation triggers cellular stress and induces the NF-κB inflammatory cascade. The NF-κB activation occurs via IκB phosphorylation and its subsequent degradation, leading to p65/p50 nuclear translocation and the transcription of pro-inflammatory genes. This process contributes to chronic inflammation, organ damage, and systemic inflammatory responses characteristic of Fabry disease.

Lysosomes are membrane-enclosed organelles containing more than 60 hydrolytic enzymes, and their role is to promote the degradation of a variety of cellular components and biological polymers such as lipids, proteins, nucleic acids, and polysaccharides [1]. When mutations occur in the genes encoding for lysosomal hydrolases or membrane transporters and give rise to what are called Lysosomal Storage Diseases (LSDs) [2], a group of inherited metabolic disorders caused by enzyme deficiencies within the lysosome result in the accumulation of undegraded substrate [3]. Defective lysosomal storage function is implicated in a broad spectrum of clinical manifestations depending on the specific substrate and site of accumulation, ranging from common diseases including neurodegenerative disease to rare lysosomal storage disorders [1,4]. Currently, more than 70 different types of LSDs have been described, and, among them, it is possible to find Pompe disease, Gaucher disease, Niemann–Pick disorders, mucopolysaccharidoses, and Anderson–Fabry disease [2,3,5]. The latter was first documented in 1898 by Johannes Fabry and William Anderson, hence the name. Anderson–Fabry disease, or Fabry disease (FD, OMIM #301500), is currently recognized as the second most common LSD after Gaucher disease [6]. It is a rare, inherited multisystem disorder caused by mutations in the alpha-galactosidase A (*GLA*) gene, located on the long arm of the X chromosome [7,8]. As an X-linked condition, affected males can only transmit the gene to their daughters, while heterozygous females have a 50% chance of passing it to both their daughters and sons [7]. Females with FD can exhibit a wide range of signs and symptoms, which may vary from being asymptomatic to displaying severe manifestations. This variability can be attributed to the phenomenon known as lyonization, which is the random inactivation of one X chromosome in each somatic cell during embryonic development [6,7]. It has been reported that female heterozygous patients with Fabry disease can have a wide range of disease severities that could develop into a mild to severe stage of disease [9]. However, there is no clear correlation between genotype and phenotype. Indeed, the phenotypic expression of FD in females is significantly influenced by the degree of X-chromosome inactivation (XCI) [10]. A deficiency in the enzyme alpha-galactosidase A (α-Gal) leads to the buildup of globotriaosylceramide (Gb3) and its deacylated form, globotriaosylsphingosine (Lyso-Gb3), in tissues and blood, resulting in multi-organ system disease that can give rise to a variety of symptoms and complications [11,12]. The activity of α-Gal in afflicted women can range from low to normal as a result of XCI and cannot be reliably diagnosed by enzyme testing, so genetic analysis is required [13,14]. It is possible to distinguish two main phenotypes of FD: the early-onset classic severe form, characterized by very low or null enzymatic activity, and the late-onset form where residual enzymatic activity is present [7]. Heterozygous females generally have milder symptoms at a later age of onset than males. The distinction between early and late forms of Fabry disease is indeed more applicable to male patients, as they are typically more severely affected due to their hemizygous genotype. In males, the classic form of Fabry disease often shows early in life with severe symptoms, while the late-onset form manifests later [15]. The reduction in enzymatic activity and the accumulation of Gb3 result in cell and organ dysfunction, particularly affecting the cardiac, renal, and nervous systems. Patients with the classical form exhibit a range of clinical manifestations, including angiokeratomas, cornea verticillata, gastrointestinal issues, acroparesthesias, and hypohidrosis, typically beginning in childhood. Cardiac, renal, and cerebrovascular involvement may occur in adulthood [7,16,17]. On the other hand, patients with late-onset phenotypes may exhibit single-organ involvement and do not have the early signs of the classical phenotype resulting in difficulties in diagnosis [16,17]. The mutations in the GLA gene may influence the clinical presentation of FD, with some mutations linked to the early-onset phenotype and others, mainly missense mutations, associated with late-onset disease [7,16]. However, due to considerable phenotypic variability among individuals with the same mutation type, establishing a clear correlation between mutation and FD phenotype is very difficult [18,19]. Furthermore, GVUS, which cannot be definitively classified as pathogenic-like classic or late-onset variants, need to be considered. Patients with these mutations often exhibit standard levels of α-Gal enzymatic activity and normal values of Lyso-Gb3. However, they simultaneously show symptoms associated with FD, adding complexity to the diagnostic process. The literature also does not allow for a clear classification, and some are often considered conflicting interpretations. Their uncertain nature may lead to challenges in establishing a definitive diagnosis and in the development of personalized treatment. Given that, resolving the diagnostic and therapeutic challenges posed by GVUS remains a critical area for further research [20,21,22]. It is supposed that the high levels of glycolipids in the cells and plasma of FD patients are not sufficient to justify the pathophysiology of this disorder. It has been found that the accumulation of Gb3 or lyso-Gb3 could trigger different cellular mechanisms that contribute to the phenotypic expression of this disease [23,24]. This may explain the different clinical manifestations in family members with *GLA* mutations, known as intra-familial phenotypic variability [25]. The accumulation of unmetabolized substrates is not merely a storage anomaly; it triggers various pathogenic cascades, such as altered lipid trafficking, endoplasmic reticulum stress, autophagy, oxidative stress, autoimmune responses, and inflammation [25,26]. These processes impair normal cellular functions, leading to multiple systemic manifestations [27]. In addition to the substrate degradation, lysosomes play also an important role in many other cellular processes, including a crucial role in normal immune system function [26,28] as phagocytosis, the release of pro-inflammatory mediators [29,30], antigen presentation, and processing [29,30,31]. As a result, pathologies altering lysosome function are consequently hypothesized to have an impact on the immune system [32,33]. There is some evidence to support the idea that distinct mechanisms of the immune system are activated in Fabry disease [34]. It has been demonstrated that the accumulation of Gb3 and lyso-Gb3 induces the Toll-like receptors (TLRs), particularly TLR4, which stimulate the production of pro-inflammatory cytokines and chemokines and inflammasome activation. The binding of glycolipids such as lyso-Gb3 to TLR4 triggers Notch-1 signaling, which subsequently activates the nuclear factor kappa B (NF-κB) pathway with the consequent production of pro-inflammatory cytokines, which give rise to both systemic and local inflammatory responses [35,36,37] (Figure 1). The lysosomal deposition of unmetabolized glycolipid substrates stimulates the activation of inflammation, and, as the stimulus cannot be eliminated, the inflammatory response is continuously activated and becomes chronic [25]. It follows that, as hypothesized by Rozenfeld et al., FD can be classified as an autoinflammatory disorder [25]. Inflammation plays a key role in the pathogenesis of FD. While this immune response is initially protective, its chronic nature inevitably contributes to the disease’s progression and complexity [27]. The autoinflammatory process may also be involved in the development of Fabry disease.

There are several mechanisms, in addition to activation of the innate immune system, whereby the ongoing accumulation of Gb3 and lyso-Gb3 have been reported to trigger inflammation [34]. Inflammation is a complex biological response of the innate immune response, which is generally nonspecific and represents the initial defense mechanisms against local infection and a variety of harmful stimuli, including pathogens, damaged cells and toxic compounds [25,38,39], and acts as a protective response by removing them and initiating the healing process [40] to restore homeostasis [41,42,43]. In physiological conditions, inflammation is an acute response, caused by the release of cytokines and other inflammatory mediators with the consequent extravasation of leukocytes into tissues, that stops once the trigger is eliminated [25]. When the inflammation continues over a long period and becomes chronic, it can occur increased cellular damages and pathogenesis that becomes unconnected from the substrate accumulation by which it was first initiated [25,44]. Furthermore, chronic inflammation is a gradual and quiet process that frequently goes unnoticed by patients until the damage and clinical consequences become untreatable. NF-κB, in addition to playing a central role in several biological processes, is the principal mediator of the inflammatory response. It induces the expression of various pro-inflammatory genes, including those encoding cytokines and chemokines, and also participates in inflammasome regulation [45]. NF-κB also controls the expression of genes that encode growth factors, cell adhesion molecules, cell cycle enzymes, and proteins involved in regulating apoptotic processes [46]. The Nuclear factor-kB family consists of five inducible transcription factors: RelA (p65), RelB, c-Rel and the precursor proteins p105 (also known as NF-κB1), and p100 (also known as NF-κB2), which are processed in p50 and p52, respectively. All these proteins shared a conserved region in the amino terminus known as the “Rel Homology Domain” (RHD), which contains sites for binding, dimerization, interaction with inhibitors (IkB), and a nuclear translocation sequence [47,48,49]. These factors mediate the transcription of target genes by binding to a specific DNA element, the kB enhancer, either as heterodimers or homodimers [45,47,48,50,51]. In the absence of stimuli, NF-κB proteins are retained in the cytoplasm by a family of inhibitory proteins, which include members of the IkB family and other related proteins characterized by ankyrin repeats [52,53]. The NF-κB activation is mediated by two key signaling pathways: the canonical and the non-canonical. Both are essential for regulating immune and inflammatory responses, despite their differing signaling mechanisms [54,55,56]. The activation of the canonical pathway occurs through the inducible degradation of IκBα, following site-specific phosphorylation by a multi-subunit IκB kinase (IKK) complex [57]. This process results in the nuclear translocation of canonical NF-κB members, primarily the p50/RelA and p50/c-Rel dimers [58,59]. The p65/p50 heterodimer is the most abundant of the NF-κB dimers [46,47]. NF-κB and the glucocorticoid receptor (GR) are physiological antagonists in the inflammation process; in fact, they are, respectively, involved in the regulation of pro- and anti-inflammatory balance. The GR, a ligand-activated transcription factor belonging to the superfamily of nuclear receptors, translocates to the nucleus after binding glucocorticoids [60]. It then regulates the expression of glucocorticoid responsive genes by binding to Glucocorticoid Responsive Elements (GREs) in specific promoter regions, acting as an anti-inflammatory agent and immunosuppressant [61,62]. The splicing of the *Rel*A gene in humans leads to a messenger, which is translated into the corresponding protein starting from the first methionine present on exon 0 with a resulting protein of 551 amino acids. Recently, a new splice variant of p65 was identified and named p65 iso5 [63]. This new isoform is characterized by the presence of a new exon, named exon −1, located upstream of the first known exon of p65 (exon 0). The presence of exon −1 induces a splicing event between exon −1 and exon 1, leading to the exclusion of exon 0 where the translation start site of p65 is contained. It follows that the p65 iso5 mRNA translation will start from the second ATG on exon 2, giving rise to a protein of 520 aa. Briefly, p65 iso5 in the presence of synthetic glucocorticoids (GCs) amplifies the anti-inflammatory response mediated by the glucocorticoid receptor. Depending on its dimerization partner, the p65 isoform can activate the promoters of specific target genes in a different way from the wild-type protein. It has been demonstrated that p65 iso5 can bind dexamethasone, indicating a distinct ability to modulate GC-mediated effects. Furthermore, the p65 iso5 protein expression is associated with inflammation-related liver diseases [63]. The distinct biochemical properties of p65 iso5, in contrast to the wild-type protein, offer valuable insights into the complex regulation of inflammation resolution. These findings strongly indicate that p65 iso5 could play a significant role in the development of inflammatory diseases, highlighting this novel protein as a potential therapeutic target. Considering earlier findings and recognizing the crucial role of comprehending the mechanisms behind NF-κB pro-inflammatory functions to improve therapeutic approaches for inflammation-related diseases, we investigated the gene expression and the protein expression level of p65 iso5 in the PBMCs of FD patients with classic and late-onset phenotypes, as well as with GVUS mutations.

## 2. Materials and Methods

### 2.1. Study Design and Population

Genetic and biochemical studies were performed at the Centre for Research and Diagnosis of Lysosomal Storage Disorders of IRIB-CNR in Palermo. Signed informed consent was obtained from patients. Our patient cohort encompassed 106 individuals (40 males and 66 females) who had been diagnosed with Fabry disease. In this study, a control group of healthy individuals (12 males and 8 females) was also included. The diagnosis of Fabry disease was established by clinical examination, reduced enzymatic activity, genetic tests, and LysoGb3. This study’s nature and purpose were explained to all volunteers, and informed consent was obtained from all participants before their involvement in this research.

### 2.2. Genetic Analysis

Peripheral blood for genetic and biochemical analyses was collected, using EDTA as an anticoagulant, and dried on absorbent paper (dried blood spot, DBS). Genomic DNA was isolated from dried blood spot using silica-coated magnetic particles in a robotic workstation designed for automated purifications of nucleic acids (Qiagen, Hilden, Germany). Mutations in the *GLA* gene were studied using Sanger sequencing. Eight primer pairs were designed to analyze eight target regions, covering the seven exons of the GLA gene, the flanking regulatory sequences, and the cryptic exon. The PCR products were purified and sequenced using an automated DNA sequencer.

### 2.3. α-Galactosidase A Activity Assay

α-galactosidase A activity assays were performed using the dried blood filter paper (DBFP) test described by Chamoles et al. [64], with some modifications (unpublished data). A spot of 10 μL of blood in a circle of paper 6 mm in diameter was placed into a 96-well plate, suitable for fluorometric assays, and incubated for 18 h at 37 °C in a thermomixer (Eppendorf, Hamburg, Germany); the reaction was terminated by the addition of 250 μL of 0.1 mol/L ethylenediamine (pH 11.4). The background fluorescence, which is not due to the specific enzyme activity, was determined for each sample, conducting another reaction in the presence of 0.14 mmol/L of 1-deoxygalactonojirimycin (DGJ, the inhibitor of alpha galactosidase A) in a citrate phosphate buffer (pH 4.5). This background was subtracted from the fluorescence of the sample. In each assay, we added positive and negative controls and a calibration curve with 4-methylumbelliferone. Normal values were >3.0 nmol/mL/h.

### 2.4. Lyso-Gb3 Determination

The determination of Lyso-Gb3 in blood was performed via tandem mass spectrometry (MS/MS) methodology, as previously described by Polo et al. [65].

### 2.5. Isolation of PBMCs in Human Samples

Blood samples from 106 Fabry patients, (mean age: 43.77 ± 19.28 years old; range: 2–87 years old) were taken for this study. Twenty healthy individuals (mean age: 41.75 ± 12.20; range: 28–64 years old) were used as controls. Peripheral blood samples were collected in Vacutainer tubes treated with ethylenediaminetetraacetic acid (EDTA) as anticoagulant. PBMC cells from whole blood were isolated by Ficoll Paque (Cytiva) gradient separation following the manufacturer’s instructions. Blood samples were diluted to a 1:2 volume ratio with PBS1X and gently layered on top Ficoll. After centrifugation at 870× *g* for 30 min with the brake off, cells were harvested and washed twice in PBS1X.

### 2.6. RNA Extraction from PBMCs and RT-PCR

Total RNA was extracted from the PBMCs by TRIzol Reagent (Invitrogen) following the manufacturer’s protocols. The RNAs were quantified with a Nanodrop (NanoDrop One-Thermo Scientific, Wilmington, DE, USA) measuring absorbance at 260 nm (A260). The RNA purity was estimated by the A260/A280 ratio and electrophoresed on agarose/formaldehyde gel for quality control of the sample. The isolated total RNA samples were reverse-transcribed using oligo (dT) primers, RNAsin RNase Inhibitor, M-MLV Reverse Transcriptase and dNTP (Promega).

### 2.7. Nested PCR

A nested PCR reaction was performed to amplify the entire p65 iso5 mRNA from the PBMCs of Fabry patients and healthy subjects. In the first reaction, specific primers were used for exon −1 (Forward Ex-1 5′-GTGACATCACCAAACTCCGCCGATC-3′) and the 3′ untranslated region (Reverse 3′UTR 5′-AGAATCCGTAAGTGCTTTTGGAGG-3′). The second reaction of the nested PCR used 5 µL (dilution 1:40) of the product of the first amplification reaction as the template, with oligonucleotides placed internal to the first primer pair on exon −1 (Forward Ex-1.1 5′-TGAAATCCCCTAAAAACAAA-3′) and exon 10 (Reverse Ex10 5′-TCTGGGGAGGGCAGGCGTCAC-3′). Nested PCR products were loaded on agarose (1%) gels.

### 2.8. Real-Time Quantitative PCR

Real-Time quantitative PCR (qPCR) for the p65 iso5 was performed using 10 μL of the ExcelTaq 2X Fast Q-PCR Master Mix (SYBR, ROX) (SMOBIO), 10 pmol/μL of the respective forward and reverse primers, 50 ng of cDNA and RNase-free H_2_O (GIBCO), reaching a final volume of 20 μL. The oligonucleotides used for qPCR were designed on exon −1/exon 1 (Table 1), as previously reported in Spinelli et al. [63] based on the gene sequences taken from the NCBI nucleotide database (GeneBank) and were validated for the absence of secondary structures, self-dimers, as well as primer efficiency and specificity.

Human β-actin gene was used as the internal control. All cDNA samples were tested in three replicates for housekeeping genes on the same 96-well PCR plate replicated in 40 cycles (95 °C for 20 s, 95 °C for 3 s, 60 °C for 30 s) to reduce possible variations in relative housekeeping genes. Non-template controls and reverse transcription controls were additionally performed. For qPCR, a StepOnePlus Real-Time PCR System (Applied Biosystems, Waltham, MA, USA; Thermo Fisher, Waltham, MA, USA) was used with 96-well PCR plates covered with MicroAmp Optical Adhesive Film (Applied Biosystems, Thermo Fisher). Data were expressed as −ΔCt in the cells of Fabry patients versus healthy controls.

### 2.9. Protein Sample Preparation

After PBMC isolation, cells were lysed in TRIzol and, consequently, to sample processing for RNA, and proteins were extracted following the manufacturer’s protocols with minor modifications in terms of protein solubilization. The cells were lysed in a lysis buffer (7 M of Urea, 2 M of Thiourea, 30 mM of CHAPS, 2% Triton ×100, 39 mM of TRIS pH 8.8, 65 mM of DTT, 1 mM of Na_3_VO_4_, 1% protease inhibitor cocktail) employing 20 min heating at 50 °C vortexing and centrifuged at 10,000× *g* at 15 °C for 10 min. The supernatant containing the total proteins has been recovered and stored at −80 °C until use. The protein concentrations were determined by Bradford assay (BioRad). To obtain the sample Cos1+p65iso5, the cells were plated in a 25 cm^2^ flask and then transfected 24 h later with a p65 iso5 expression vector.

### 2.10. Western Blot Analysis

Protein samples (15 or 30 µg) were separated in a 10% polyacrylamide-SDS gel. Proteins were transferred to a nitrocellulose membrane (Amersham Protran 0.45 NC nitrocellulose Western blotting membranes GE Healthcare) overnight at 4 °C, in agitation in a Tris/glycine/methanol 20% buffer at 50 V. The non-specific sites of the membranes were blocked with 5% bovine serum albumin (BSA) in Tris-Buffered Saline containing 0.1% Tween-20 (TBS-T buffer) with gentle agitation for 30 min at room temperature after the membranes were incubated at room temperature in a fresh solution of 5% BSA in TBS-T containing the primary antibody NF-κB p65 (D14E12) Rabbit mAb (1:5000) (Cell Signaling, Danvers, MA, USA) for 2 h at room temperature. After incubation, membranes were washed three times with TBS-T (7 min each) and re-incubated with Alexa Fluor 680 goat anti- rabbit IgG 1:10,000 (Invitrogen, Waltham, MA, USA) diluted in 5% BSA in TBS-T for 1 h at room temperature. The membranes were washed three times with TBS-T (5 min each) and once with TBS. Membranes were scanned and analyzed using the Odyssey infrared imaging system (LI-COR Biosciences, Lincoln, NE, USA) and Odyssey 3.0 imaging software. The band intensities of the experimental target and housekeeping protein were quantified by densitometric analysis considering the normalization factor and the level of protein expression is normalized with the mean of the controls. Quantitative analysis of protein bands was performed using ImageJ analysis software.

### 2.11. Statistical Analysis

Data are expressed as mean ± standard deviation (SD) for continuous variables with normal distribution. For Real-Time PCR and Western blot analysis, the statistically significant difference was determined by *t*-test with GraphPad Prism Software (GraphPad Software, Inc., San Diego, CA, USA). *p* value < 0.05 was considered statistically significant.

## 3. Results

### 3.1. Fabry Disease Patient Population Overview

**Figure 2:**
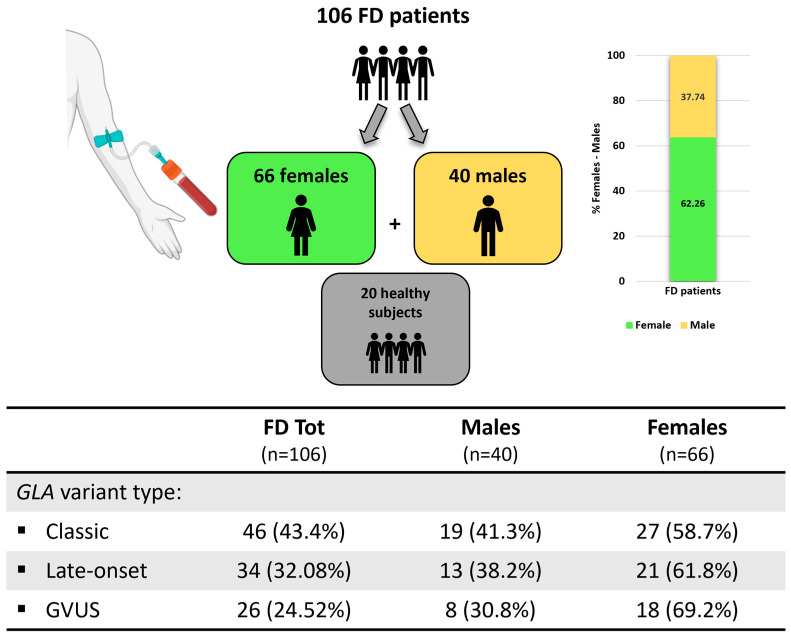
Overview of Fabry disease population included in this study. 126 subjects were included, 106 affected by FD and 20 healthy subjects used as controls.

Our study encompassed 126 subjects, whose 20 healthy volunteers were used as a control group and 106 with FD divided as follows: 46 with classic phenotype, 34 with late-onset phenotype and 26 with a genetic variant of unknown significance. In the total FD group, 43.4% were represented by the classic variant, followed by late-onset and GVUS variants with, respectively, 32.08% and 24.52% of the patients. In the classic group, 41.3% were males and 58.7% females. In the late-onset group, 38.2% were represented by males and 61.8% females. In the GVUS group, 30.8% were males, and 69.2% were females (Figure 2).

All the 106 subjects included in this study exhibited clinical symptoms typically associated with Fabry disease, such as angiokeratoma, ventricular hypertrophy, acroparesthesia, intolerance to heat or cold, renal insufficiency, etc., or were relatives of previously diagnosed patients. The average age of the 106 patients included in this study was 43.77 ± 19.28 years, and Table 2 reports more detailed information according to sex and genetic variants.

**Figure 3:**
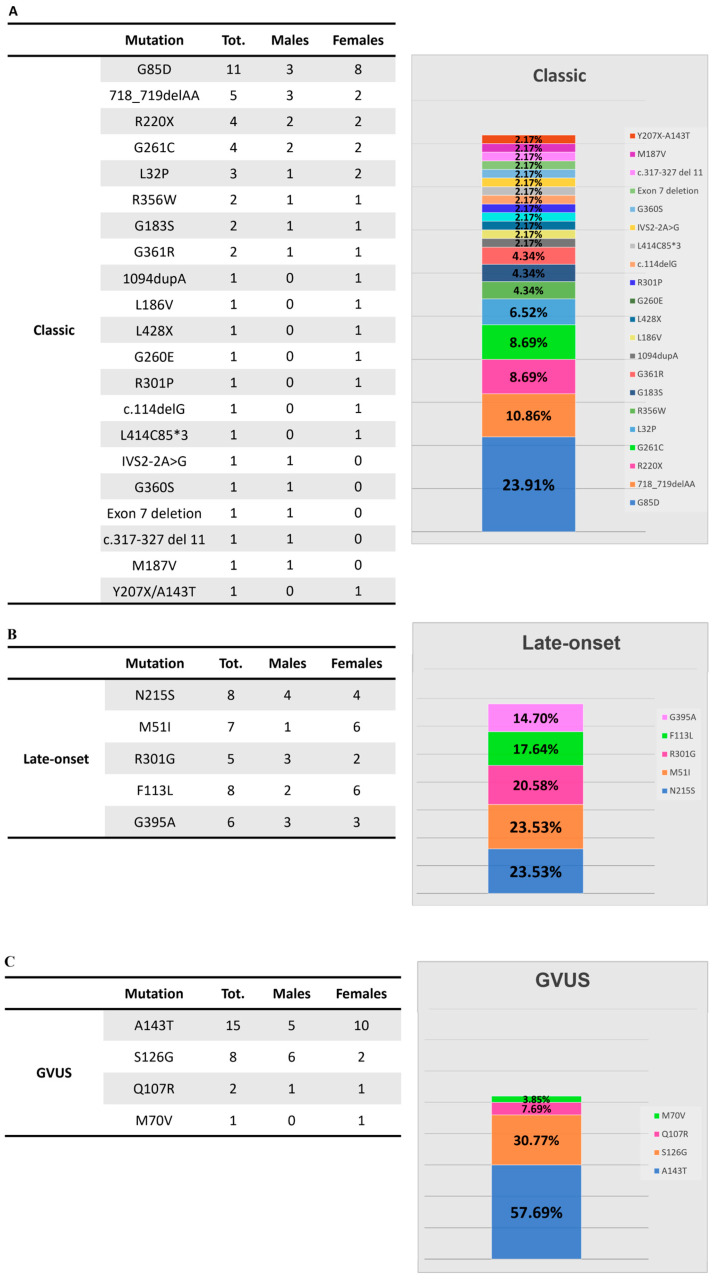
Subjects included in this study with classic (**A**), late-onset (**B**) and GVUS (**C**) mutations in the *GLA* gene.

As described above, the group with the classic variant consists of 46 individuals, where the most represented mutation is the G85D with 11 individuals (23.9%). The second most represented mutation is 718_719delAA followed by R220X and G261C with four subjects each. The other mutations are represented in Figure 3A and divided by sex. The group of patients with the late-onset variant is composed of 34 individuals. The most represented mutations are N215S and F113L followed by M51I, R301G, and G395A (Figure 3B). The group of GVUS consisted of 26 individuals, 15 of whom had the A143T mutation followed by S126G with 8 subjects (more detailed information is given in Figure 3C). Among patients of each group, symptoms experienced vary according to the phenotype and, even in individuals sharing the same type of mutation, a significant phenotypic variability can be observed. The women present a wide variability in the severity of their phenotype mainly due to the random X-chromosome inactivation in each of their cells (Lyon hypothesis) [49]. In Table 3, the prevalence of various symptoms can be observed—cardiac, renal, and neurological manifestations—among males and females across the three groups of patients (classic, late-onset, and GVUS) included in our study. Patients of the classic group (n = 46) typically present early symptoms as acroparesthesia, hypohidrosis, cornea verticillata, heat and cold intolerance, recurrent fever, angiokeratomas, microalbuminuria/proteinuria, and gastrointestinal symptoms followed by more severe cardiac, renal, and neurological manifestations. Among cardiac manifestations, most common in the male patients, there are left-ventricular hypertrophy (LVH) and heart failure. Renal manifestations are most prevalent in the females’ group (70.4%), whereas neurological manifestations are more common in males (73.7%). In the late-onset group (n = 34), without the early signs of the classical phenotype, cardiac symptoms are the most frequent in both males (69.2%) and females (61.9%) followed by neurological and renal manifestations. In the GVUS group (n = 26), neurological manifestations are most common in males (75%), while renal manifestations are predominant in females (72.2%).

Furthermore, we also reported the values of α-galactosidase A enzyme activity and the dosage of the substrate Lyso-Gb3 in the blood of all patients with classic, late-onset and GVUS variants, and the differences in male and in female patients (Table 4).

### 3.2. Identification and Evaluation of the mRNA Expression Levels of p65 iso5 in PBMCs of Fabry Patients

In Fabry disease, the phenotype should be determined by the genotype, although this correlation is not always clearly defined [66,67]. As reported in the literature and as observed in patients with many other genetic diseases, the FD phenotypes can also vary significantly in subjects that have the same mutation on the *GLA* gene [68,69]. In previous work, we identified a new isoform of p65, a protein of the NF-κB complex, called p65 iso5, whose expression is associated with inflammation-related diseases [63]. These findings, along with other data present in the literature about the pro- and anti-inflammatory role functions attributed to NF-κB [70], suggest that p65 iso5 could be implied in the fine regulation of inflammatory response [63]. In Fabry disease, inflammation becomes chronically stimulated due to continuously producing glycolipid deposits [71]. Therefore, we decided to evaluate the possible variation in p65 iso5 mRNA expression and protein in the PBMCs of FD patients compared to healthy subjects. To amplify the entire transcript of p65 iso5 in PBMCs of FD subjects, several nested PCRs were performed, using specific primers for exon −1 and exon 10 located, respectively, in the 5′ and the 3′ untranslated region.

**Figure 4:**
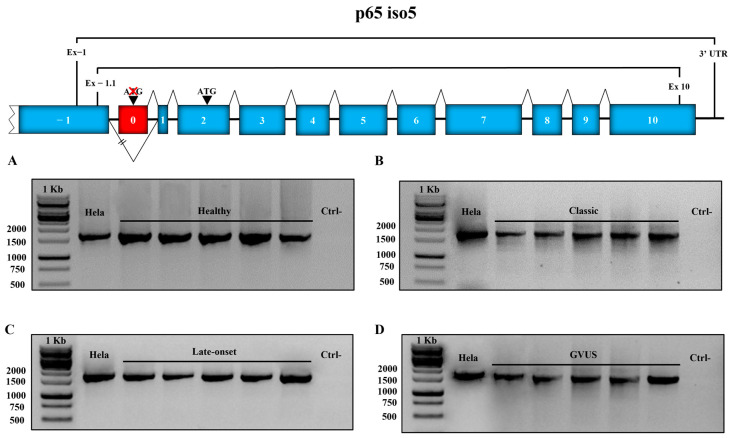
Nested RT-PCR of p65 iso5 in PBMCs of healthy subjects and FD patients. The first-round PCR reaction was executed using specific oligonucleotides for the exon −1 and 3′ UTR (untranslated region). For the second round PCR (performed on first round dilution 1:40), oligonucleotides were used for exon −1 and exon 10. The − symbol indicates the absence of reverse transcriptase in the reaction (representative of each sample). The PCR reactions were performed on healthy subjects (**A**), patients with classic (**B**), late onset (**C**), and GVUS mutations (**D**).

Here, we show a representative number of PCRs performed on a representative number of samples of each genetic variant and healthy subjects. Furthermore, PCRs were performed on cDNA extracted from HeLa cells as an additional control. The expected dimension of the transcript was 1694 bp, and the sequence analysis was also performed. As shown in Figure 4, the analysis of PCR products on agarose gel showed that the transcript of the expected dimension is present in all the analyzed samples belonging to classic (Figure 4B), late-onset (Figure 4C), and GVUS (Figure 4D) groups as well as healthy subjects (Figure 4A).

### 3.3. mRNA Expression Levels of p65 iso5 in PBMCs from Fabry Patients

**Figure 5:**
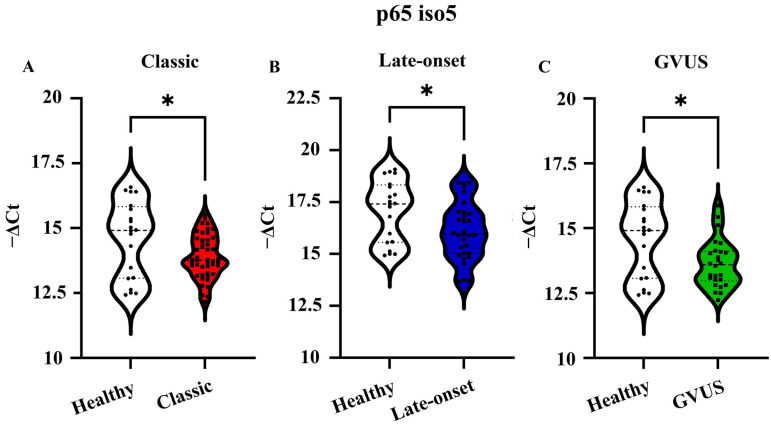
mRNA relative quantity of p65 iso5 in PBMCs of subjects affected by FD. The Ct and median measuring gene expression levels of p65 iso5 in PBMCs of FD subjects with (**A**) classic in red (46 subjects); (**B**) late-onset in blue (34 subjects) and (**C**) in green GVUS (26 subjects) variants. The p65 iso5 mRNA is significantly down-regulated in patients with FD and with respect to the healthy control. Data are presented as mean ± SEM. * *p* < 0.05.

To study the p65 iso5 gene expression profile, the qPCR analysis was performed on a group constituted by a total of 126 samples divided as follows: 106 FD patients and 20 healthy subjects used as the control. In the first group, there were 46 subjects with the classic variant, 34 with late-onset, and 26 with GVUS. Following the analysis of the data considering males and females together, dividing them according to the genetic variant, the results showed that the p65 iso5 mRNA is significantly down-regulated in classic, late-onset, and GVUS variants compared to healthy subjects (Figure 5).

**Figure 6:**
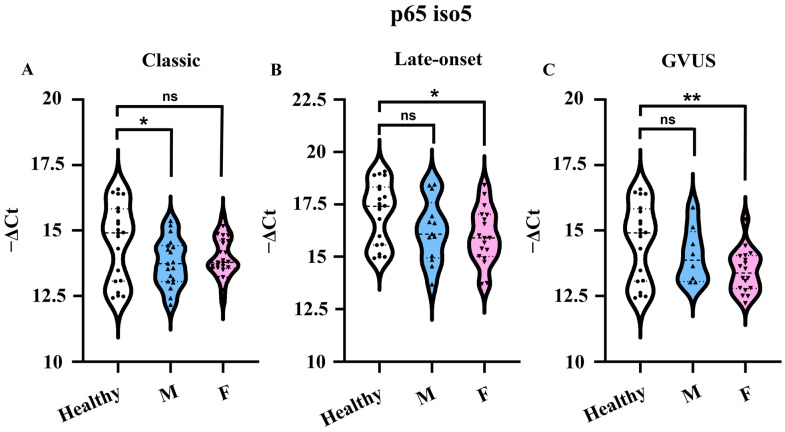
mRNA relative quantity of p65 iso5 in PBMCs of FD patients divided by sex. The Ct and median measuring gene expression levels of p65 iso5 in PBMCs of FD subjects with (**A**) classic: 19 males (light-blue) and 27 females (pink); (**B**) late-onset: 13 males (light-blue) and 21 females (pink), and (**C**) GVUS: 8 males (light-blue) and 18 females (pink). The p65 iso5 mRNA is significantly down-regulated in male patients with the classic variant and in females with late onset and GVUS variants, with respect to the healthy control. Data are presented as mean ± SEM. * *p* < 0.05 and ** *p* < 0.01.

Furthermore, we decided to analyze the data considering the difference between males and females in correlation with the classic variant, atypical, and GVUS. The results revealed a sex-based difference. In the male population, p65 iso5 mRNA is significantly down-regulated in the classic variant, whereas, in the female population, the data are significant for the late-onset and GVUS variants (Figure 6). It is known that different mutations can lead to the same phenotype, but, at the same time, the same mutation can cause different phenotypes with consequent difficulty in the correlation between the genotype and phenotype [72]. These results could underline, once again, the clinical variability that characterizes Fabry’s disease, even within a single family and with intersex variability encompassing the allelic heterogeneity of mutations with the consequent symptom onset and the variable disease severity [73,74]. Furthermore, it should also be taken into account that the random X chromosome inactivation, also called lyonization, in female FD patients can give rise to significant clinical variability [75].

### 3.4. Analysis of p65 iso5 Protein Expression in PBMCs of Fabry Patients

**Figure 7:**
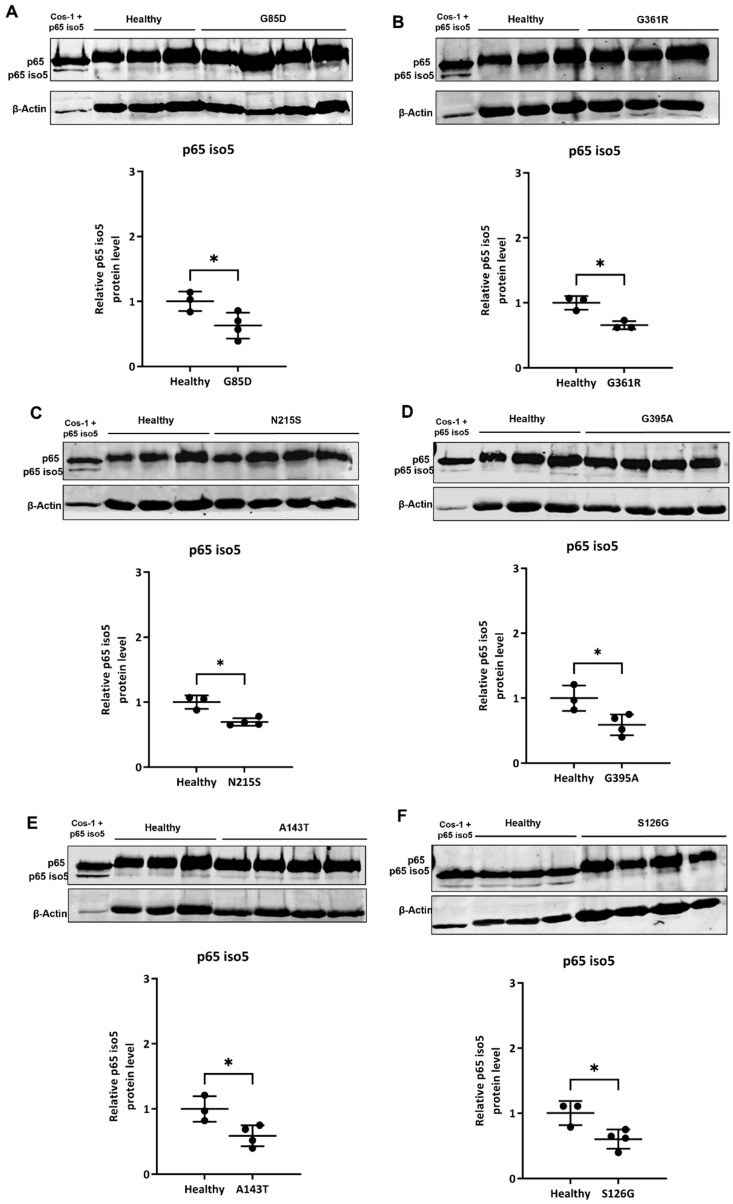
Different p65 iso5 protein expression in FD subjects compared to control. The p65 iso5 relative protein quantity was evaluated in the PBMCs of FD subjects with classic variant: G85D (**A**) and G361R (**B**); late-onset variant: N215S (**C**) and G395A (**D**); GVUS variant: (**E**) A143T and (**F**) S126G, with respect to healthy control. The data are representative of several Western blot assessments. The band intensities of the experimental target and housekeeping protein were quantified by densitometric analysis considering the normalization factor. The level of protein expression is normalized with the mean of the controls (n = 3), with each bar representing the standard deviation * *p* < 0.05.

The link between the TLR4 activation and nuclear translocation of NF-κB, with the consequent transcription of genes involved in immune response and inflammation, results in the chronic inflammation observed in FD [76]. Considering the variability of the p65 iso5 expression, depending on the inflammatory pathology examined, in this study, we thought to evaluate the relationship between mRNA levels and p65 iso5 protein expressions in a representative sample of PBMCs of FD patients, with classic (Figure 7A,B, see Figures S1 and S2 for entire membrane images), late-onset (Figure 7C,D, see Figures S3 and S4 for entire membrane images), and GVUS (Figure 7E,F, see Figures S5 and S6 for entire membrane images) variants by Western blot analysis. Furthermore, the expression of the p65 iso5 protein was analyzed, as control, in the Cos-1 cell transfected with the p65 iso5 expression vector. For the p65 iso5 protein detection, an antibody was used, directed against an epitope of the C-terminal region and able to detect even the wild-type protein. The results, consistent with the mRNA relative quantity of p65 iso5, showed a significant decrease in the relative p65 iso5 protein level in patients with FD with respect to healthy control (Figure 7, see Figures S1–S6 for entire membrane images). Further investigations are probably needed for a larger number of samples. This could help to identify a correlation between the different p65 iso5 protein expressions among the genetic variants of FD in comparison to healthy subjects.

### 3.5. Focus on Late Onset Phenotype: F113L Mutation

**Figure 8:**
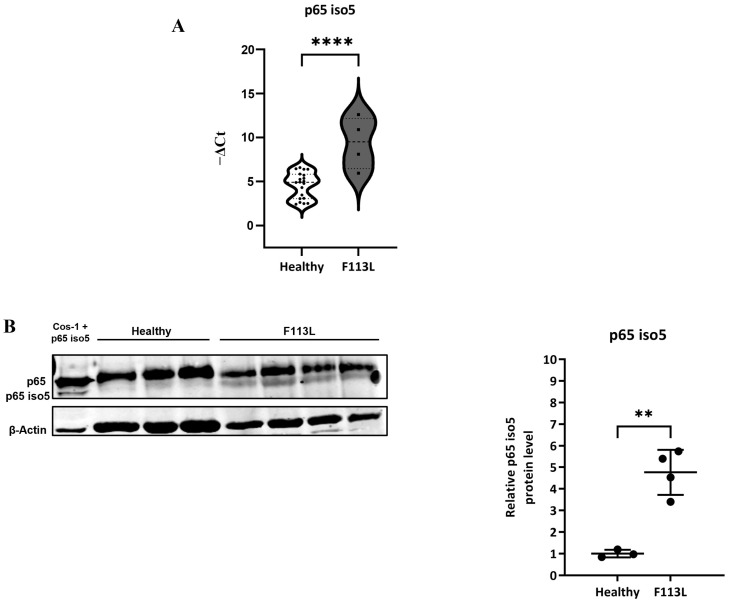
p65 iso5 mRNA and p65 iso5 protein relative quantity in the PBMCs of 4 FD subjects with F113L mutation: (**A**) p65 iso5 mRNA relative quantity evaluated in the PBMCs of 4 subjects with F113L mutation (in grey 1 male, 3 females), with respect to the healthy control (n = 3). Data are presented as mean ± SEM. **** *p* < 0.0001. (**B**) p65 iso5 relative protein quantity evaluated in the PBMCs of 4 subjects with F113L mutation (1 male, 3 females), with respect to healthy control. The data are representative of several Western blot assessments. The band intensities of the experimental target and housekeeping protein were quantified by densitometric analysis considering the normalization factor, and the level of protein expression is normalized with the mean of the controls (n = 3), with each bar representing the standard deviation ** *p* < 0.01.

The missense mutation c.337T>C (p.F113L) is a pathogenic mutation of the *GLA* gene, related to a late-onset phenotype, that results in a misfolded and unstable enzyme at the neutral pH within the endoplasmic reticulum, leading to its degradation [77]. Although this mutation is primarily linked to significant cardiac involvement (affecting 40.8% of patients) [78], the history of FD associated with this mutation remains actually unclear. It has also been documented in a 46-year-old male who exhibited severe renal impairment and sensorineural deafness without any cardiac issues [79], and in a family presenting a renal phenotype [80]. By analyzing data related to mRNA levels and protein expression of p65 iso5, it was observed that some samples, belonging to the group with the F113L mutation, behaved differently than others with the same mutation. Particularly, four samples (one male and three females), showed an up-regulation in p65 iso5 mRNA levels (Figure 8A) and an increase in relative p65 iso5 protein levels (Figure 8B, see Figure S7 for entire membrane image). This difference in the p65 iso5 mRNA level could support the hypothesis that the classification of this variant is not exclusively cardiac as reported in a previous study [78], underlying the underestimates of its systemic involvement and different phenotype among patients with the same mutation.

## 4. Discussion

Inflammation plays an important role in the pathological progression of organ disease and is mainly regulated by the NF-κB, MAPK, and JAK-STAT pathways. The dysregulation in one or more of these can be involved in the onset of inflammation-associated disease [38]. Inflammation is known to be involved in FD, but the precise molecular and cellular mechanisms linking the intracellular accumulation of glycolipids to the inflammatory process and subsequent organ damage are not yet fully understood [8,25]. The presence of missense or nonsense mutations in the *GLA* gene is responsible for α-GalA enzyme deficiency. This causes accumulation of the glycosphingolipid globotriaosylceramide (Gb3) and its deacylated derivate globotriaosylsphingosine (lyso-Gb3) in cells, leading to lysosomal storage disease associated with Fabry, characterized by chronic inflammation, and multiorgan disease [81]. The accumulation of Gb3 leads to the activation of TLR4 with the consequent activation of NOTCH1 signaling and the NF-κB pathway. The consequence is the production of pro-inflammatory cytokines and a chronic inflammatory state, which contributes to generate cellular and organ damage [35,37]. A comprehensive understanding of the inflammatory response and the molecular mechanisms involved in Fabry disease is essential. In this study, we aim to contribute to a deeper understanding of the NF-κB mediated inflammatory process in the FD. Our previous findings identified a new isoform of p65, a protein of the NF-κB complex, called p65 iso5 [63]. This protein was previously shown to bind dexamethasone and to regulate the glucocorticoid response in a different way from the wild-type protein. The p65 iso5 protein expression was found to be associated with inflammation-related liver diseases, and p65 iso5 mRNA was overexpressed in the PBMCs of COVID-19 patients, suggesting a key player in the regulation of inflammation [63]. Our results highlight a different expression of p65 iso5 in FD subjects compared to healthy subjects, showing that the p65 iso5 mRNA level and protein are significantly down-regulated in all FD groups (classic, late-onset, and GVUS) compared to healthy controls. This suggests a potential role of p65 iso5 in the inflammatory response of FD subjects. In addition, our results showed sex-specific differences in the p65 iso5 mRNA expression. In males, a significant down-regulation of p65 iso5 mRNA was observed in the classic variant, while, in females, significant down-regulation was found in the late-onset and GVUS variants. These differences highlight the clinical variability in FD and suggests that alternative mechanisms could be due to sex and mutation type. It should be noted that, given the chronic and progressive nature of Fabry disease, a longitudinal study design would be ideal for investigating the long-term progression of clinical symptoms and to track changes in individual patients over time. However, we believe that our findings could set the bases for future research that can provide a more comprehensive understanding of Fabry disease. A Western blot analysis of p65 iso5 protein levels in the PBMCs of FD patients showed a significant decrease, consistent with the mRNA expression data obtained by qPCR analysis. This supports the hypothesis that a reduction in p65 iso5 expression could be associated with FD and its inflammatory chronic state. Within the late-onset group, particularly those with the F113L mutation, some samples presented a p65 iso5 mRNA up-regulation and an increased protein level. This suggests that the F113L mutation, commonly classified as cardiac variant, may have broader systemic implications, indicating the heterogeneity of FD even within specific mutations. In fact, patients with the same mutation can exhibit alternative phenotypes, which may be influenced by factors such as environmental stimuli, additional genetic variations, and differential X-inactivation in females due to lyonization.

## 5. Conclusions

Our results, therefore, highlight the significant clinical variability in FD, emphasizing that the genotype–phenotype correlation in FD is complex and unclear. The variability in p65 iso5 expression suggests that this isoform may play a crucial role in modulating the chronic inflammatory response in Fabry disease. Although much progress has been made in the pathophysiology comprehension, many aspects remain unclear. The therapies currently used, such as enzyme replacement and pharmacological chaperone, have made much progress in recent years, but the investigation of new therapies like substrate reduction, gene therapy, and anti-inflammatory drugs introduces new opportunities [27]. Targeting p65 iso5 or its regulatory pathways could offer new therapeutic strategies to reduce chronic inflammation in FD. Considering the different p65 iso5 expression among different Fabry phenotypes and sexes, further investigations would be needed to understand the contribution of this protein in FD pathogenesis. This could lead to the realization of personalized treatment approaches, taking into account individual genetic backgrounds. In conclusion, FD due to α-Gal A enzyme deficiency is characterized by advanced organ damage and inflammation that plays a very important role in pathogenesis. A deeper understanding of the inflammatory response in FD could lead to new therapeutic strategies. The different expression of p65 iso5 in FD patients compared to healthy controls suggests its potential role in the pathogenesis of the disease. Our results, underline the importance of comprehending the different molecular mechanisms at the base of chronic inflammation in Fabry disease. These findings contribute to increasing the knowledge of FD and highlight the need for further research to explore the therapeutic potential of targeting inflammatory pathways, particularly those involving NF-κB and p65 iso5.

## Figures and Tables

**Table 1:** Oligonucleotides used in qPCR analysis for p65 iso5.

Target	Forward Primer	Reverse Primer	Dimension (bp)
**p65 iso5**	AGCCCTGGCTTTGCTCCAGACC	CCGGGAAGATGAGGGGGAAC	118

**Table 2:** α-Galactosidase A activity and LysoGb3 blood accumulation in subjects with classic, late-onset and GVUS variants. Enzyme activity is measured in nmol/mL/h (normal values > 3 nmol/mL/h). Lyso-Gb3 is measured in nmol/L (normal values are 0.1–2.3 nmol/L).

**FD Tot**	**Patients**	**Male**	**Female**
Patients	106	40	66
Average α-Gal A activity	7.57	3.76	9.89
Average LysoGb3	11.22	23.95	3.84
**Classic**	**Patients**	**Male**	**Female**
Patients	46	19	27
Average α-Gal A activity	4.7	0.55	7.62
Average LysoGb3	22.74	44.63	7.34
**Late-onset**	**Patients**	**Male**	**Female**
Patients	34	13	21
Average α-Gal A activity	6.53	2.88	8.80
Average LysoGb3	3.04	5.63	1.43
**GVUS**	**Patients**	**Male**	**Female**
Patients	26	8	18
Average α-Gal A activity	14.03	12.80	14.57
Average LysoGb3	1.52	1.83	1.38

**Table 3:** Frequency of cardiac, renal, and neurological symptoms according to sex in patients with classic, late-onset, and GVUS variants.

	Symptoms	Male (n = 19)	Female (n = 27)
	Cardiac manifestations	8 (42.1 %)	8 (29.6 %)
**Classic** (n = 46)	Renal manifestations	9 (47.4 %)	19 (70.4 %)
	Neurological manifestations	14 (73.7 %)	13 (48.1 %)
	**Symptoms**	**Male** (n = 13)	**Female** (n = 21)
	Cardiac manifestations	9 (69.2 %)	13 (61.9 %)
**Late-onset** (n = 34)	Renal manifestations	6 (46.1 %)	5 (23.8 %)
	Neurological manifestations	6 (46.1 %)	11 (52.4 %)
	**Symptoms**	**Male** (n = 8)	**Female** (n = 18)
	Cardiac manifestations	1 (12.5 %)	4 (22.2 %)
**GVUS** (n = 26)	Renal manifestations	4 (50 %)	13 (72.2 %)
	Neurological manifestations	6 (75 %)	10 (55.5 %)

**Table 4:** Average age of all patients included in this study with Fabry disease (n = 106) and healthy subjects (n = 20) considered in this study.

	Healthy (n = 20)	Classic (n = 46)	Late-Onset (n = 34)	GVUS (n = 26)
	Age, yo	Age, yo	Age, yo	Age, yo
	41.8 ± 12.20	43.9 ± 19.70	45.6 ± 20.70	41.2 ± 16.90
**Males**	47.17 ± 12.89	33.84 ± 19.17	52 ± 22.92	43.38 ± 13.93
**Females**	33.63 ± 4.14	51 ± 17.06	41.57 ± 18.69	40.22 ± 18.34

## Data Availability

The original contributions presented in this study are included in the article/Supplementary Material. Further inquiries can be directed to the corresponding author.

## Supplementary Materials

The following supporting information can be downloaded at https://www.mdpi.com/article/10.3390/cells14030230/s1, Figure S1: Entire membrane representing samples from healthy subjects and samples from FD patients with G85D mutation; Figure S2: Entire membrane representing samples from healthy subjects and samples from FD patients with G361R mutation; Figure S3: Entire membrane representing samples from healthy subjects and samples from FD patients with N215S mutation; Figure S4: Entire membrane representing samples from healthy subjects and samples from FD patients with G395A mutation; Figure S5: Entire membrane representing samples from healthy subjects and samples from FD patients with A143T mutation; Figure S6: Entire membrane representing samples from healthy subjects and samples from FD patients with S126G mutation; and Figure S7: Entire membrane representing samples from healthy subjects and samples from FD patients with F113L mutation.
