# Supplementary material for: Fabry Disease and Inflammation: Potential Role of p65 iso5, an Isoform of the NF-κB Complex
Source: Cells. 2025 Feb 6;14(3):230. doi: 10.3390/cells14030230 (PMC11817417; doi:10.3390/cells14030230)
Supplement: Supplementary file 1 [file cells-14-00230-s001.zip › cells-3391507-supplementary.pdf]

Supplementary Materials

Fabry Disease and Inflammation: Potential Role of p65 iso5, an Isoform of the NF-κB Complex

Giuseppa Biddeci, Gaetano Spinelli, Paolo Colomba, Giovanni Duro, Monia Anania, Daniele Francofonte and Francesco Di Blasi *

Institute for Biomedical Research and Innovation, National Research Council of Italy (IRIB-CNR), Via Ugo La Malfa 153, 90146 Palermo, Italy;

* Correspondence: francesco.diblasi@irib.cnr.it; Tel.: +39-0916809514

Figure S1

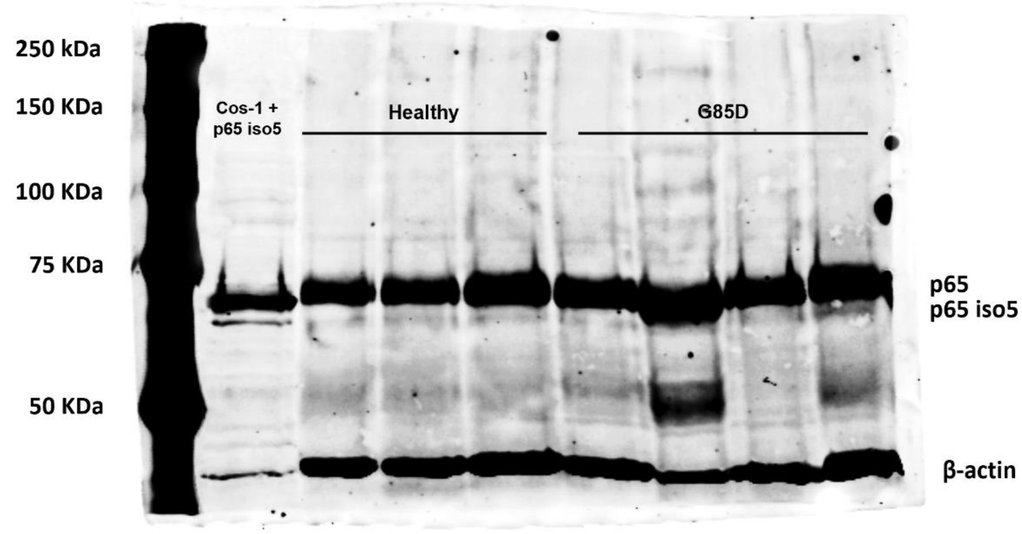

Figure S1. Entire membrane representing sample from healthy subjects and sample from FD patients with G85D mutation. The non-specific sites of the membranes were blocked with 5% bovine serum albumin (BSA) in Tris-Buffered Saline containing 0.1% Tween-20 (TBS-T buffer) with gentle agitation for 30 min at room temperature. After the membranes were incubated at room temperature in a fresh solution of 5% BSA in TBS-T containing the primary antibody NF-κB p65 (D14E12) Rabbit mAb (1:5000) (Cell Signaling) for 2 h at room temperature. After incubation, membranes were washed three times with TBS-T (7 min each) and re-incubated with Alexa Fluor 680 goat anti- rabbit IgG 1:10000 (Invitrogen) diluted in 5% BSA in TBS-T for 1 h at room temperature. The membranes were washed three times with TBS-T (5 min each), and once with TBS. Membranes were scanned and analyzed using the Odyssey infrared imaging system (LI-COR Biosciences, Lincoln, NE, USA) and Odyssey 3.0 imaging software.

Figure S2

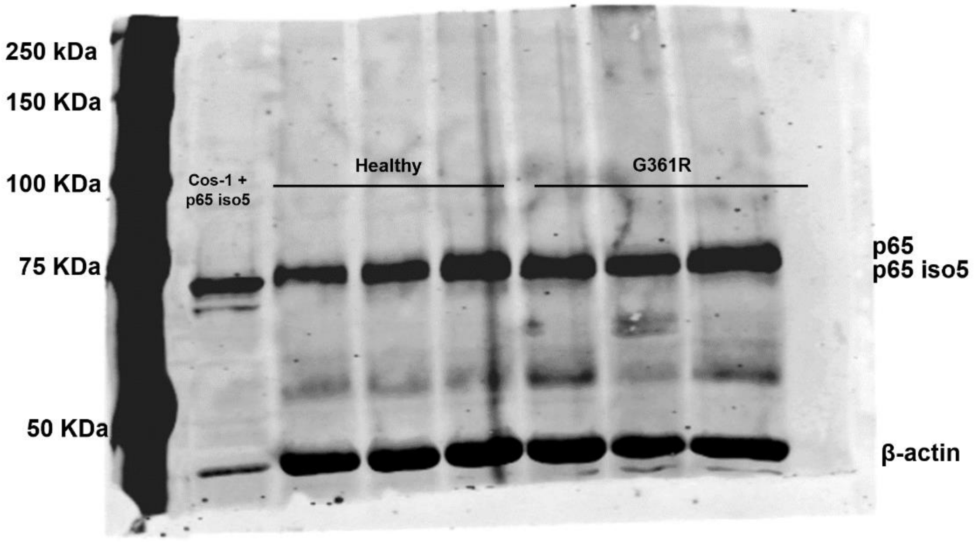

Figure S2. Entire membrane representing sample from healthy subjects and sample from FD patients with G361R mutation. The non-specific sites of the membranes were blocked with 5% bovine serum albumin (BSA) in Tris-Buffered Saline containing 0.1% Tween-20 (TBS-T buffer) with gentle agitation for 30 min at room temperature. After the membranes were incubated at room temperature in a fresh solution of 5% BSA in TBS-T containing the primary antibody NF-κB p65 (D14E12) Rabbit mAb (1:5000) (Cell Signaling) for 2 h at room temperature. After incubation, membranes were washed three times with TBS-T (7 min each) and re-incubated with Alexa Fluor 680 goat anti- rabbit IgG 1:10000 (Invitrogen) diluted in 5% BSA in TBS-T for 1 h at room temperature. The membranes were washed three times with TBS-T (5 min each), and once with TBS. Membranes were scanned and analyzed using the Odyssey infrared imaging system (LI-COR Biosciences, Lincoln, NE, USA) and Odyssey 3.0 imaging software.

Figure S3

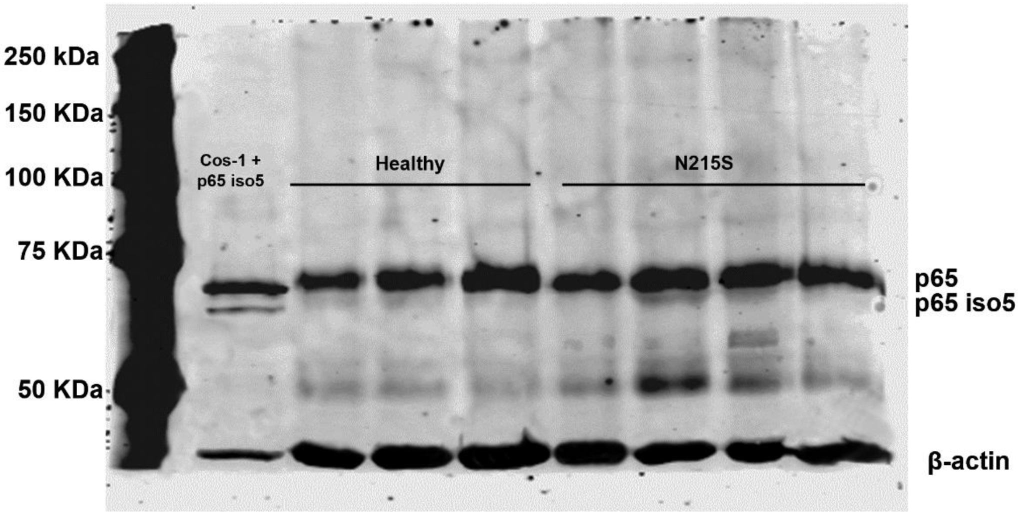

Figure S3. Entire membrane representing sample from healthy subjects and sample from FD patients with N215S mutation. The non-specific sites of the membranes were blocked with 5% bovine serum albumin (BSA) in Tris-Buffered Saline containing 0.1% Tween-20 (TBS-T buffer) with gentle agitation for 30 min at room temperature. After the membranes were incubated at room temperature in a fresh solution of 5% BSA in TBS-T containing the primary antibody NF-κB p65 (D14E12) Rabbit mAb (1:5000) (Cell Signaling) for 2 h at room temperature. After incubation, membranes were washed three times with TBS-T (7 min each) and re-incubated with Alexa Fluor 680 goat anti- rabbit IgG 1:10000 (Invitrogen) diluted in 5% BSA in TBS-T for 1 h at room temperature. The membranes were washed three times with TBS-T (5 min each), and once with TBS. Membranes were scanned and analyzed using the Odyssey infrared imaging system (LI-COR Biosciences, Lincoln, NE, USA) and Odyssey 3.0 imaging software.

Figure S4

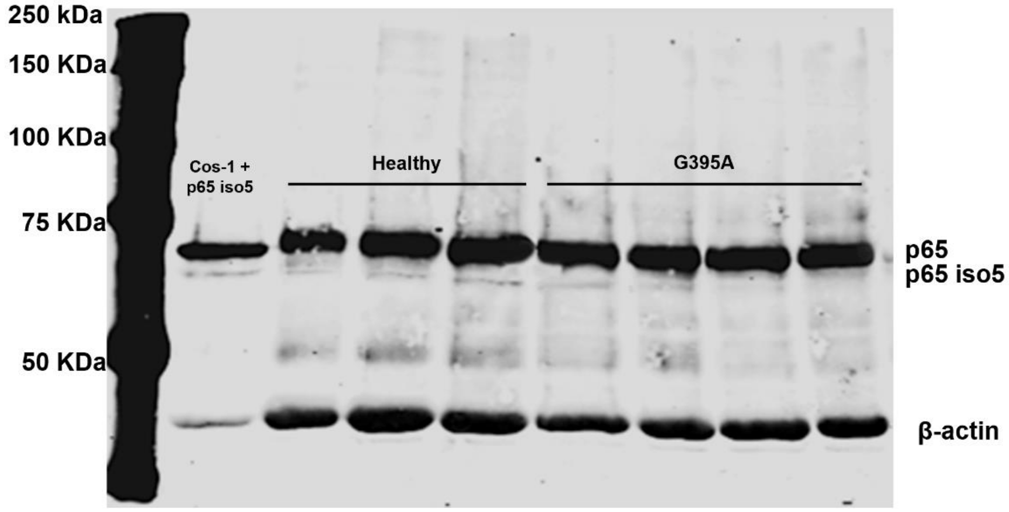

Figure S4. Entire membrane representing sample from healthy subjects and sample from FD patients with G395A mutation. The non-specific sites of the membranes were blocked with 5% bovine serum albumin (BSA) in Tris-Buffered Saline containing 0.1% Tween-20 (TBS-T buffer) with gentle agitation for 30 min at room temperature. After the membranes were incubated at room temperature in a fresh solution of 5% BSA in TBS-T containing the primary antibody NF-κB p65 (D14E12) Rabbit mAb (1:5000) (Cell Signaling) for 2 h at room temperature. After incubation, membranes were washed three times with TBS-T (7 min each) and re-incubated with Alexa Fluor 680 goat anti- rabbit IgG 1:10000 (Invitrogen) diluted in 5% BSA in TBS-T for 1 h at room temperature. The membranes were washed three times with TBS-T (5 min each), and once with TBS. Membranes were scanned and analyzed using the Odyssey infrared imaging system (LI-COR Biosciences, Lincoln, NE, USA) and Odyssey 3.0 imaging software.

Figure S5

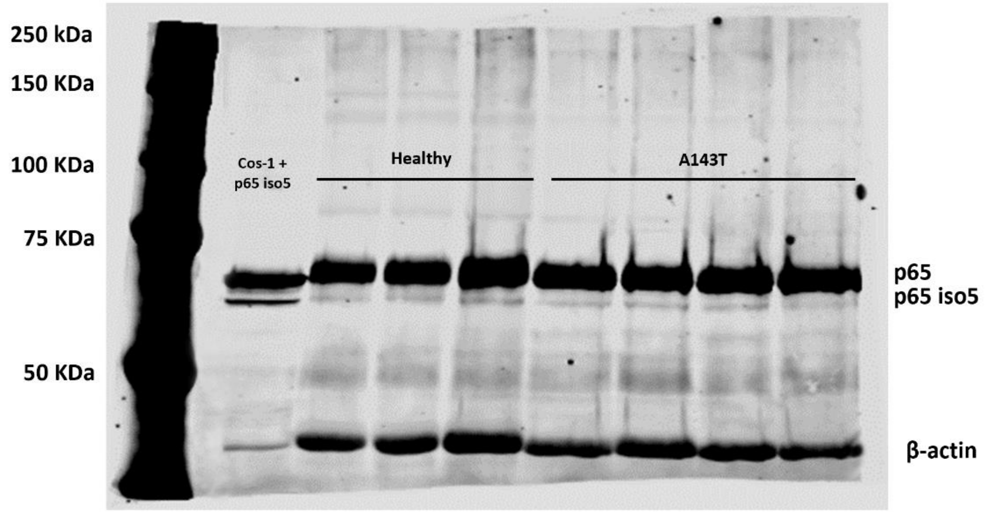

Figure S5. Entire membrane representing sample from healthy subjects and sample from FD patients with A143T mutation. The non-specific sites of the membranes were blocked with 5% bovine serum albumin (BSA) in Tris-Buffered Saline containing 0.1% Tween-20 (TBS-T buffer) with gentle agitation for 30 min at room temperature. After the membranes were incubated at room temperature in a fresh solution of 5% BSA in TBS-T containing the primary antibody NF-κB p65 (D14E12) Rabbit mAb (1:5000) (Cell Signaling) for 2 h at room temperature. After incubation, membranes were washed three times with TBS-T (7 min each) and re-incubated with Alexa Fluor 680 goat anti- rabbit IgG 1:10000 (Invitrogen) diluted in 5% BSA in TBS-T for 1 h at room temperature. The membranes were washed three times with TBS-T (5 min each), and once with TBS. Membranes were scanned and analyzed using the Odyssey infrared imaging system (LI-COR Biosciences, Lincoln, NE, USA) and Odyssey 3.0 imaging software.

Figure S6

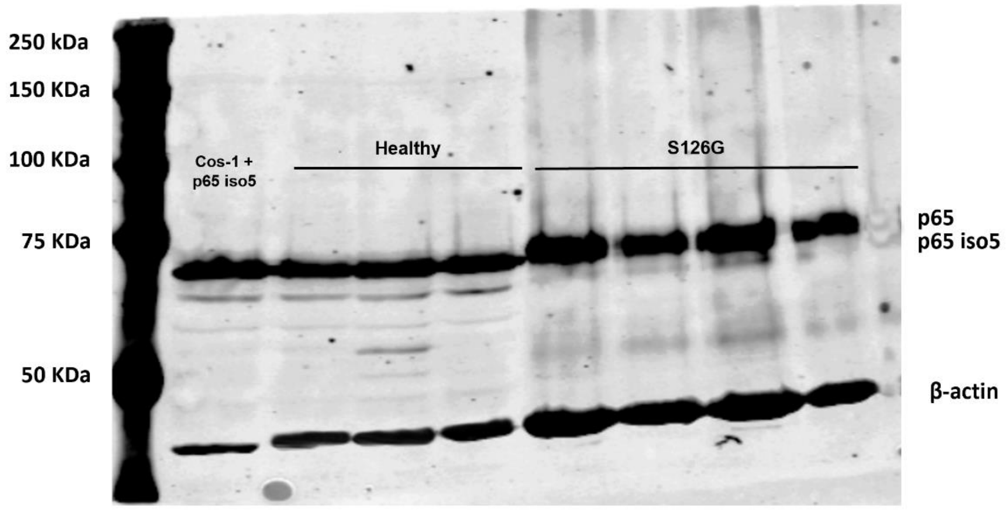

Figure S6. Entire membrane representing sample from healthy subjects and sample from FD patients with S126G mutation. The non-specific sites of the membranes were blocked with 5% bovine serum albumin (BSA) in Tris-Buffered Saline containing 0.1% Tween-20 (TBS-T buffer) with gentle agitation for 30 min at room temperature. After the membranes were incubated at room temperature in a fresh solution of 5% BSA in TBS-T containing the primary antibody NF-κB p65 (D14E12) Rabbit mAb (1:5000) (Cell Signaling) for 2 h at room temperature. After incubation, membranes were washed three times with TBS-T (7 min each) and re-incubated with Alexa Fluor 680 goat anti- rabbit IgG 1:10000 (Invitrogen) diluted in 5% BSA in TBS-T for 1 h at room temperature. The membranes were washed three times with TBS-T (5 min each), and once with TBS. Membranes were scanned and analyzed using the Odyssey infrared imaging system (LI-COR Biosciences, Lincoln, NE, USA) and Odyssey 3.0 imaging software.

Figure S7

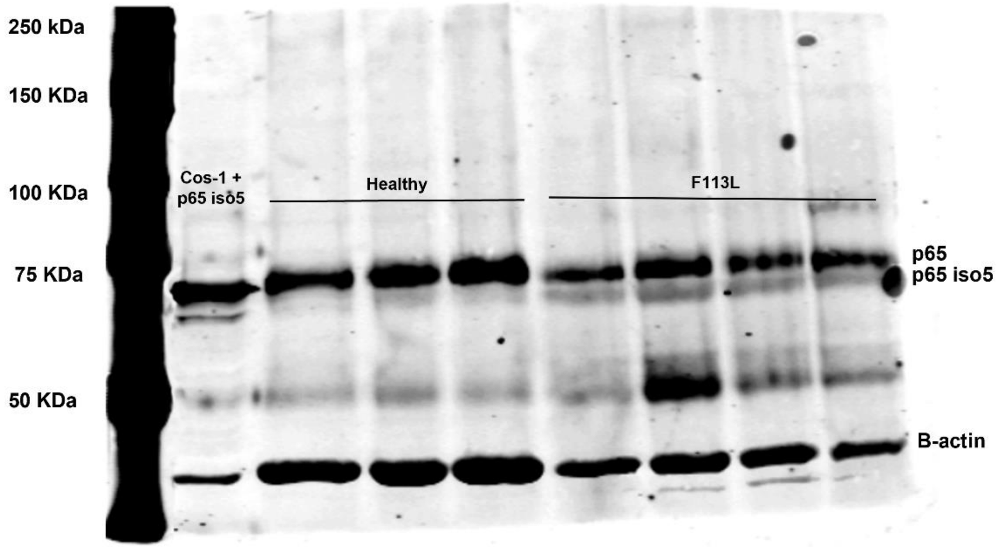

Figure S7. Entire membrane representing sample from healthy subjects and sample from FD patients with F113L mutation. The non-specific sites of the membranes were blocked with 5% bovine serum albumin (BSA) in Tris-Buffered Saline containing 0.1% Tween-20 (TBS-T buffer) with gentle agitation for 30 min at room temperature. After the membranes were incubated at room temperature in a fresh solution of 5% BSA in TBS-T containing the primary antibody NF-κB p65 (D14E12) Rabbit mAb (1:5000) (Cell Signaling) for 2 h at room temperature. After incubation, membranes were washed three times with TBS-T (7 min each) and re-incubated with Alexa Fluor 680 goat anti- rabbit IgG 1:10000 (Invitrogen) diluted in 5% BSA in TBS-T for 1 h at room temperature. The membranes were washed three times with TBS-T (5 min each), and once with TBS. Membranes were scanned and analyzed using the Odyssey infrared imaging system (LI-COR Biosciences, Lincoln, NE, USA) and Odyssey 3.0 imaging software.
